# The dimensionality and latent structure of mental health difficulties and wellbeing in early adolescence

**DOI:** 10.1371/journal.pone.0213018

**Published:** 2019-02-26

**Authors:** Louise Black, Margarita Panayiotou, Neil Humphrey

**Affiliations:** Manchester Institute of Education, University of Manchester, Manchester, United Kingdom; Chiba Daigaku, JAPAN

## Abstract

Research with adults and older adolescents suggests a general factor may underlie both mental health difficulties and wellbeing. However, the classical bifactor model commonly used to demonstrate this general trait has recently been criticised when a unidimensional structure is not supported. Furthermore, research is lacking in this area with children and early adolescents. We present confirmatory factor analysis models to explore the structure of psychopathology and wellbeing in early adolescents, using secondary data from a large U.K. sample (*N* = 1982). A simple correlated factors structure fitted the data well and revealed that wellbeing was just as related to internalising as this was to externalising symptoms. The classical bifactor solution also fitted the data well but was rejected as the general factor explained only 55% of the total common variance. *S*-1 models were therefore used to explore general covariance in a more robust way, and revealed that a general internalising distress factor could play an important role in all item responses. Gender and income differences in mental health were also explored through invariance testing and correlations. Our findings demonstrate the importance of considering mental health difficulties and wellbeing items together, and suggestions are made for how their correspondence could be controlled for.

## Introduction

Both mental ill health and positive wellbeing in young people are associated with outcomes such as academic attainment and social functioning [1–5], as well as demographic and environmental correlates [6–14]. The majority of mental health problems have first onset in adolescence [15], and can result in significant disability [6, 8, 9]. Furthermore, it is widely agreed that adolescence, ranging from ages 10–24, is critical to functioning in later life [16–18], while recent evidence suggests young people’s mental health may be deteriorating [6, 12].

Despite this clear need to understand the form of mental health, particularly in young people, its conceptualisation and measurement have been inconsistent. A historic focus on disorder remains the basis for measurement [19], even though the absence of disorder symptoms consistently fails to fully explain wellbeing in young people [1–5, 7, 13, 14]. The limitations of categorical diagnoses are also becoming increasingly clear, with criticisms focussing predominantly on stigmatisation via poorly evidenced medical models [19], and a lack of validity for discrete disorders [20, 21]. For instance, hyperactivity disorders have been criticised as pathologising typical and expected behaviour in children and adolescents, particularly boys [22], and studies have repeatedly failed to discern groups experiencing one externalising disorder without other comorbid problems [23–25]. Symptom-level and hierarchical approaches, on the other hand, are emerging as useful ways to understand structure, risk and comorbidity in mental health difficulties. Such approaches have demonstrated consistent covariance between symptoms, cutting across traditional disorder taxonomies [20, 26–32]. In fact, not only is there strong evidence of general covariance between symptoms of mental health, longitudinal research (from birth to midlife) suggests that experiencing symptoms of mental disorder is the norm, with only a small minority remaining completely symptom-free over time [33]. This supports the current shift in understanding, in which taxonomic approaches to mental disease classification are being rejected. Continuous dimensional frameworks are instead being adopted and encouraged, to reflect evidence that mental health symptoms seem to be extreme and distressing variations in typical processes rather than indicative of categorical diagnoses [34].

While *dual-factor* approaches have sought to gain a more comprehensive view of child and adolescent mental health by capitalising on the benefits of wellbeing measures [2], they too have typically resorted to simplistic categorical approaches. Though a moderate relationship between psychopathology and wellbeing has been consistently demonstrated [35–38], a focus has emerged which has emphasised their dissociation, forcing participants into one of four categories [1–5, 13, 14]. At either extreme, these are content and free of symptoms (*flourishing*), and dissatisfied and suffering symptoms (*languishing*). Also included, however, are the more surprising groups of individuals who are symptom-free and dissatisfied, and satisfied but symptomatic. This approach has demonstrated the important finding that absence of symptoms is not synonymous with the presence of wellbeing. However, it distracts from the known association between the two constructs, and finding that the majority of participants are straightforwardly either flourishing or languishing [1–5, 13, 14]. Nevertheless, wellbeing approaches do not appear to suffer from the outdated biases outlined above, and in young people there is also strong correspondence between different instruments and wellbeing subtypes, suggesting strong construct validity [10]. Given the association of mental health difficulties and wellbeing, the need for continuous approaches to mental health, and the relative strengths of wellbeing measures, there is therefore an opportunity to consider these outcomes together as part of a comprehensive structure.

Despite this, robust methods interrogating the measurement structure of wellbeing and mental health difficulties in early adolescence have yet to be employed, despite the existence of theoretical frameworks such as *complete mental health*, the *two-continua* approach, or the *dual-factor model* [2, 38, 39]. The current study addresses this major gap, building on research with adults and older adolescents [38, 40, 41].

### Mental health difficulties and wellbeing

Wellbeing is typically considered to comprise positive (cognitive) evaluations of life, positive affect and the absence of negative affect [42]. These three aspects are typically considered to form hedonic wellbeing, while eudaimonic wellbeing captures aspects beyond pleasure, reflecting how well a person feels they align with their own values and ideals [43]. In young people, these different approaches to wellbeing have been shown to be highly related [10].

The present analysis draws on instruments designed for general population screening and will therefore focus on internalising and externalising symptoms. Though this means not all disorders and symptom-types are covered, this approach builds on previous research [7], provides insight into the two most common forms of mental health difficulties in childhood [8, 9], and is supported by evidence that broad internalising and externalising spectra can explain covariance across disorders [26].

Internalising is typically considered to include depressive and anxious type disorders and is therefore concerned with somatic, worry and sadness symptoms [26, 44]. There is, therefore, some conceptual crossover between this aspect of mental health difficulties and wellbeing, given that they are each is concerned with happiness or unhappiness. This can be seen in measures such as the General Health Questionnaire 12 (GHQ-12), which is sometimes considered to be a symptom measure, and sometimes a wellbeing instrument capturing negative affect [40, 45].

In children, externalising symptoms and disorders typically include conduct and attentional problems [46, 47]. Given the controversy surrounding attentional problems mentioned above, the current study focuses particularly on conduct problems. Though externalising symptoms often share comorbidity with internal distress symptoms, when considered alone these are behavioural and related to disinhibition [44].

### Gender differences in child and adolescent mental health

The prevalence of disorders between genders is complex in each developmental period. Between ages 6 and 11 boys are up to twice as likely to suffer from severe mental health difficulties, but levels of internalising symptoms are similar [7, 8, 48]. However, between 11 and 14, girls are substantially more likely to suffer from internalising problems [6, 49]. Bifactor modelling has also yielded inconsistent results: While some research has suggested a general mental health factor was not associated with gender in early adolescence [28], a study with slightly older participants suggested it was [41]. The expression of mental health is therefore linked to gender in a complex way at the beginning of adolescence (around age 11), and warrants further investigation.

Wellbeing also shows consistent complex differences for gender, varying significantly by domain [10, 11]. Typically, girls show higher satisfaction with school and social relationships, while boys are happier with their appearance [11, 12]. Overall, wellbeing is higher for boys in some countries and for girls in others [11]. In the U.K., child and adolescent boys were shown to have higher overall happiness [12]. From a unidimensional perspective, this is incongruent with the finding in the same country that boys are at greater risk of mental health difficulties [48]. However, it perhaps echoes the finding that U.K. adolescent girls are at particular risk of depression [6, 49]. The complexity of gender relationships with mental health difficulties and wellbeing challenges assumptions of unipolarity, and suggests empirical evidence of their structure is needed.

### Family income differences in child and adolescent mental health

Though country-level economic factors show no or very little association with children and adolescents’ wellbeing or mental health difficulties, household-level income is significantly associated with these outcomes [6, 10, 11, 48, 50]. While patterns for income are more straightforward than for gender, with children from poorer backgrounds reporting greater mental health difficulties and lower wellbeing, the extent to which income explains each outcome is quite different. Family income consistently more strongly predicts variability in mental health difficulties than wellbeing [6, 10, 11, 48, 50]. The existence of this relationship for both outcomes in varying strength, suggests their composite structure may provide insight into the role of income for mental health.

### Problems with the existing dual-factor approach

When mental health difficulties and wellbeing are analysed independently (i.e. any covariance is not accounted for), they do appear to be somewhat distinct. For instance, longitudinal research suggests that, even among the minority who never experience mental disorder, over 20% have been found to report low life satisfaction [33]. Similarly, the two constructs have been found to have a discrete set of correlates, as well as some shared predictors in early adolescence [7]. It remains unclear, however, to what extent items for each construct overlap and tap similar dimensions. For instance, while Patalay et al. [7] aggregated internalising and externalising symptoms (likely only moderately correlated; see [47]), and then found the corresponding coefficient between mental health difficulties and wellbeing to be only -.20, Kinderman et al. [51] treated wellbeing and internalising psychopathology as related latent factors, and these were correlated at -.82. The conceptual overlap between internalising and wellbeing alluded to above may explain this discrepancy between correlations since though both referred to outcomes as mental ill health, Kinderman et al. [51] included only depression and anxiety.

Given that mental health difficulties and wellbeing are known to be correlated, [37, 38], it seems illogical not to control for this association. Furthermore, since results are likely biased, already suggested by Patalay and Fitzsimons’[7] surprisingly low correlation between the two constructs and dimensionality is assumed rather than tested, conclusions based on analyses ignoring the association of mental health difficulties and wellbeing should be treated with caution.

### Problems with existing approaches to modelling mental health

The definitions above make clear that mental health difficulties represent a broad range of symptoms, some of which intuitively relate to wellbeing, and that these constructs show complex relationships with gender and income. Complex measurement models are already common in mental health research since high rates of comorbidity and correlations between items have led researchers to model symptoms or disorders together through bifactor structures, termed psychopathology or *p*-factor models [27]. These models have been used to argue for a general transdiagnostic factor and two studies have extended these to include wellbeing [40, 41]. Despite appropriately controlling for wellbeing, these studies have focused on older samples and age generalisability cannot be assumed [6, 10, 48]. These studies also have theoretical and methodological problems leaving many questions unanswered. For instance, the study by Böhnke et al. [40] was restricted since the measure used for mental health difficulties (the GHQ-12) has been argued by some to mainly capture negative affect [45]. Therefore the finding by Böhnke et al. [40] of a strong general factor explained almost entirely by GHQ-12 indicators is arguably unsurprising, since this measure could be expected to strongly mirror wellbeing instruments [10, 45].

While Böhnke et al. [40] studied adults in the general population, St Clair et al. [41] aimed to understand the structure of mental health in a sample of older adolescents and young adults. While symptom measures were included, these tended to be old, based on categorical diagnoses, or poorly validated [52–55], and self-esteem was also included as a measure of positive mental health with no clear theoretical justification. This is therefore at odds with contemporary spectra approaches [26], and may explain why an arguably uninterpretable result emerged: The best fitting model was a bifactor solution, but items did not always load on both general and specific factors, some loadings were low and even reversed on specific factors, and crossloadings seemed to be allowed, such that wellbeing and self-esteem items were allowed to load on a shared positive factor as well as two separate specific factors. Eid et al. [56] point out that such problematic solutions can arise where bifactor models are misapplied, while the questionable choice of measures, unsupported by theory is likely to have contributed to the results outlined above. There is, therefore, a clear need to study the complex structure of mental health in adolescents using more appropriate measures.

Beyond these specific problems with dual-factor bifactor studies, there has recently been a great deal of criticism of bifactor modelling more generally, which the current study aims to address. Firstly, where there are correlations between all indicators, as is the case in mental health models, a general factor which accounts for this covariance will always occur, even where this pattern of covariance arises for another reason, such as network structures, where one symptom leads to another [57]. Secondly, bifactor structures are highly parameterised and tend to overfit the data such that sample and measure complexity (e.g. cross loadings and correlated residuals) can be absorbed by the general factor, making the bifactor structure apparently better fitting even when this is not the case [58]. Thirdly, though evaluating competing models is important to avoid selecting a model based on close fit alone, when others may be viable or better, model comparison between correlated factors, second-order and bifactor solutions as is typically conducted could lead to false conclusions [57–59]. While these structures have substantially different interpretations, they are mathematically very close and sometimes even equivalent (depending on the number of factors). As a result, differences may not be attributable to superior structure, but instead be an artefact of the sample, unmodeled complexity or an alternative explanation for covariance such as *mutualism* in which problems co-occur [57–59]. Relative fit of such models must therefore be interpreted with caution.

**Fig 1:**
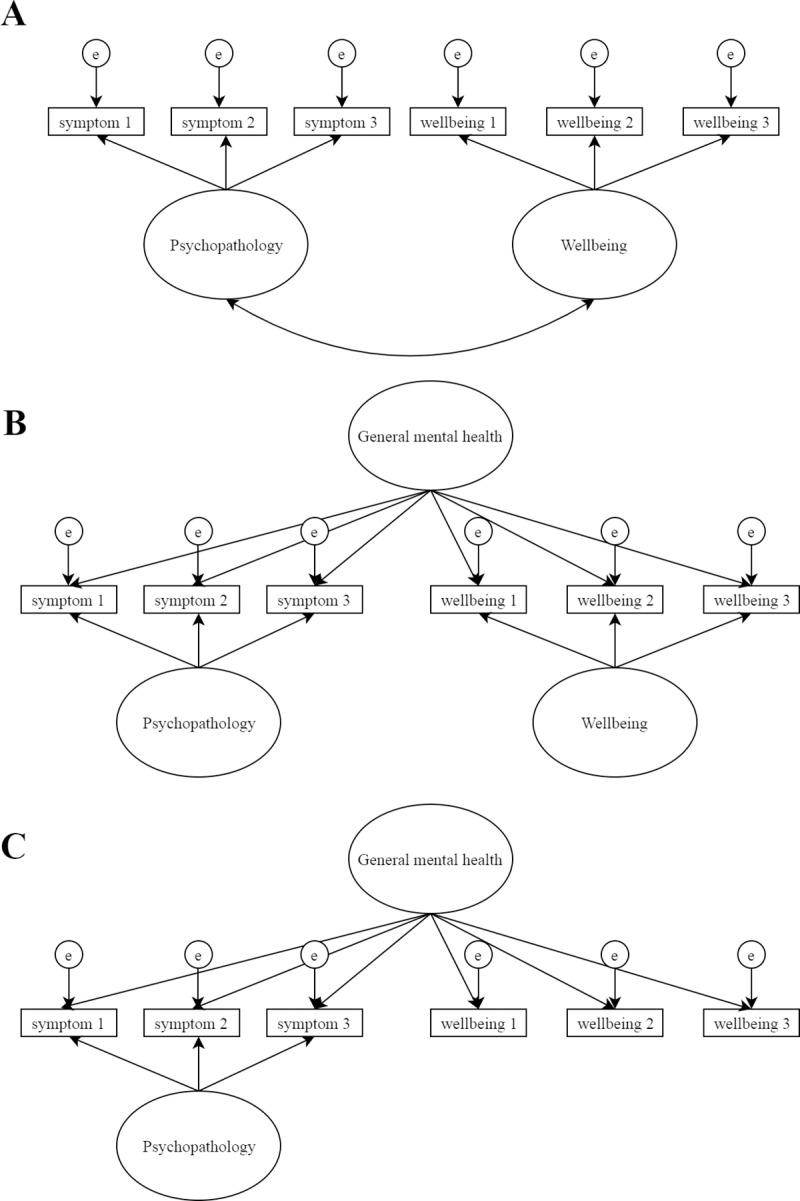
Confirmatory factor analysis model examples. (A) Correlated factors model. (B) Classical bifactor model. (C) *S*-1 model.

Recent criticisms have also proposed that the classical bifactor model (see Fig 1B) is not psychometrically well defined, since a single source of variability (the participants) is used to define a dual decomposition of a single score into two random variables, which ought to each have a distinct source of randomness [56]. This means that latent general and specific factors are unrelated while simultaneously being a function of the true score of the same indicators. Where these specific factors have substantial variance and salient loadings, these are therefore uninterpretable since they represent constructs that are wholly orthogonal to each other and the general factor, while this general factor simultaneously represents shared covariance [56, 60]. If we consider the general factor to represent liability for all symptoms, the residual specific factors must represent something wholly unrelated to the symptoms captured by the general factor [60]. On the other hand, if we consider a specific internalising factor to represent specific depressive, somatic and anxious symptomology, we must assume that the general factor does not include these in the same way. Given that both general and specific factors are generated from the same responses to the same item set, it is impossible to substantively distinguish these orthogonal true score variables as the constraints of the bifactor model require [56].

In order to estimate a meaningful general factor that captures the covariance of all items, one specific factor can be removed [56]. This allows the general factor to become a function of the true score of the items with no specific factor, so that it can become well defined psychometrically as a random variable. The general factor in this model, known as *S*-1 (see Fig 1C), however, has a slightly different interpretation. For instance, if the specific wellbeing factor is removed (*S*-1_wellbeing_), the general factor represents general wellbeing accounting for the covariance of this construct with internalising and externalising items. The specific internalising and externalising factors, on the other hand, would represent the residual variance not explained in these items by the general wellbeing domain. We argue that this model should be considered, not only because it is statistically more robust than the classical bifactor model, but also because it provides an opportunity to generate an interpretable measurement structure in the presence of general covariance but not essential unidimensionality.

Despite such criticisms, some argue bifactor models can be successfully used when essential unidimensionality is supported, such that the specific factors represent noise (e.g. method factors) [59, 60]. Such a structure was found for mental health difficulties and wellbeing in adults [40], suggesting that this should be tested in adolescence (despite the potential noise introduced by GHQ-12 noted above). Furthermore, bifactor models provide a platform to examine dimensionality via a robust method, the Explained Common Variance (ECV) index [61–63]. Though the question of dimensionality has underpinned much dual-factor research, this has yet to be statistically explored. However, for the reasons described above, and despite common practice [28, 41], we suggest that bifactor structures should not be accepted and interpreted merely based on model fit, especially when unidimensionality is not supported.

It has also been recently pointed out that measurement structures, such as bifactor models, should not be interpreted as evidence of broader construct validity, beyond measures employed [60]. The purpose of this study, however, is to demonstrate an example of models and methods needed, given that mental health difficulties and wellbeing are routinely used together as outcomes in adolescent research [2–5, 13]. We therefore aim to provide evidence of their measurement structure so that bias through failing to account for covariance, can be avoided, rather than to present a definitive structure.

### The current study

On the basis of the evidence reviewed above, several predictions were made. Firstly, latent wellbeing would be correlated with latent mental health difficulties factors, particularly internalising, at moderate levels (hypothesis 1). This hypothesis was operationalised in a correlated factors model (see Fig 1A). Secondly, we predicted that a classical bifactor solution (see Fig 1B) would fit the data well, but that this would not be essentially unidimensional as found by Böhnke et al. [40], since we used more clearly dissociated measures, and research with adolescents has also suggested multidimensionality (hypothesis 2) [41]. Thirdly, if hypotheses one and two were supported, we predicted that an *S*-1_wellbeing_ model (see Fig 1C) would provide a useful and robust structure to account for the covariance of mental health difficulties and wellbeing (hypothesis 3). This model would provide an indication of wellbeing corrected for symptoms. Finally, given that group differences have been noted across gender and income for both outcomes, we explored invariance and associations for the strongest model, based on a balance of psychometric rigor, interpretability and fit (hypothesis 4).

## Method

We conducted secondary analysis of baseline data from an evaluation of locally developed interventions designed to prevent mental health problems in young people from 12 areas of England (HeadStart) [64]. The University College London Research Ethics Committee granted ethical approval, and parental consent was given for early adolescents to complete the secure online surveys during their usual school day. Teachers read out an information sheet to pupils before these were completed. This emphasised pupils’ confidentiality and their right to withdraw.

### Participants

A total of 1982 pupils in their final year of primary education (1051 male, 53%) were drawn from 59 schools in England. Pupils’ age ranged between 10.75 and 12.25 (*M* = 11.21, *SD* = .30). The sample was not drawn to be representative since it reflected the areas participating in the HeadStart programme. As such, statements of special educational needs were below average (1.3% compared to the national average of 2.8%), while those with registered additional needs not meeting the threshold for a statement was above the national average (21.7% compared to 15.4%) [65]. The percentage of participants from white, non-ethnic minority backgrounds was also slightly above the national average for primary schools (74% compared to 70%) [66], while the number of those exposed to a language at home other than English was similar (20% compared to 19%) [66]. In terms of deprivation, 24% of participants were eligible for free school meals (FSM) when data were collected. This is above the national average of 15.6% [66], but typical of U.K. early adolescents’ mental research in schools [67].

### Measures

Self-report measures (see S1 Appendix) were used since at age 11 these are a valid indication of early adolescents’ internal perspectives [68]. Though externalising symptoms can be more accurately reported by a parent or teacher, internalising and wellbeing symptoms are considered to be more reliable from the child’s perspective [68]. Given that informant type may have an impact on the modelling structure and therefore act as a confound, the limitation of self-report for externalising was seen to be outweighed by the strength of using a single informant in the specific analysis conducted.

#### Mental health difficulties

Mental health difficulties was measured through the Me and My School (M&MS; also referred to as Me and My Feelings) questionnaire, which consists of 10 internalising, and six externalising items [69]. This measure was designed to provide a similar screening function to the Strengths and Difficulties Questionnaire [70], but for a younger age range. Participants responded *never*, *sometimes* or *always* (coded one to three) to brief statements (e.g. “I worry a lot”). Possible scores therefore ranged from 10–30 for internalising and 6–18 for externalising, assuming no missing responses. M&MS has been found to be psychometrically robust, with good internal consistency (in 11–12 year-olds, externalising α = .80, internalising α = .77); concurrent validity, *r* = .67 - .70, for equivalent, and *r* = .22–24 for non-equivalent subscales of the Strengths and Difficulties Questionnaire; and good known-groups validity between clinical and non-clinical populations [71]. M&MS contains one reverse-coded item in the externalising subscale (item 14 “I am calm”).

#### Wellbeing

Wellbeing was measured by the four-item Child Outcome Rating Scale (CORS) [72]. Four aspects (me, school, family and everything) were responded to by clicking on a smooth line between a happy and sad face. For online administration, this line was measured from 0–100, but then divided by 10 for analysis to match the paper version and facilitate model convergence. Possible scores therefore ranged between 0–10 for each item. CORS has been found to be psychometrically robust with good internal consistency (α = .84), test-retest reliability (*r* = .60), and concurrent validity (care-taker CORS, *r* = .63, care-taker Youth Outcome Questionnaire, *r* = -.43)[72]. These researchers also found good responsiveness and known-groups validity between clinical and non-clinical samples.

#### Family income

Pupil FSM eligibility is captured in a number of ways in England [73]. In the current study, data were used on whether pupils had *ever* been eligible for FSM, rather than their *current* status, since transitions in and out of poverty as well as persistent and current poverty, have all been shown to be associated with child and adolescent mental health [50]. Of the sample, 43% (*N =* 860) had ever been eligible for FSM.

### Procedure

Survey data were collected in schools in spring 2015 through a secure online portal and subsequently matched to individual socio-demographic characteristics drawn from the National Pupil Database.

### Statistical analysis

Confirmatory factor analysis (CFA) was conducted using Weighted Least Squares with Means and Variance adjustment (WLSMV) in Mplus 8.1. One exception to this was the CFA of the CORS instrument, for which robust maximum likelihood was used since all items were continuous. WLSMV was selected to account for the categorical nature of the M&MS measure [74], handle the substantial floor effects associated with screening measures [75], and because this estimator has been shown to produce minimal bias with clustered data [76]. In addition, correlated residuals, which are better handled by WLSMV [77], were of particular interest in the current study given the tendency of the classical bifactor model to absorb unmodeled complexity of this kind [58]. Finally, WLSMV is recommended where there are a large number of variables and factors, and sample size is large [77], as was the case in the current study.

Chi-square statistics are reported but not used to judge fit given their known sensitivity to sample size. The Comparative Fit Index (CFI), Tucker Lewis Index (TLI) and Root Mean Square Error of Approximation (RMSEA), and its 90% confidence interval (CI) are reported to indicate model fit, with values close to .95 for CFI and TLI, and .06 for RMSEA, typically interpreted as good fit [78]. However, given the overfitting problems associated with bifactor solutions, these indices were interpreted alongside the psychometric rigor of each model as well as other indices such as the ECV.

#### Evaluation of error variances

Given the problems with not modelling correlated systematic error where this is indicated by modification indices and theoretically supported [58, 59], this was investigated in all instruments and solutions before final models were estimated. Individual CFAs of each instrument were therefore conducted in addition to the models shown in Fig 1, so that systematic error could be evaluated here as well. The evaluation of each instrument at this stage also allowed assessment of how well factors were indicated by items, via loadings. In addition to this we calculated Cronbach’s α as basic description of subscale reliability to further ensure all items were appropriate for subsequent analysis.

While in a strict sense bifactor modeling assumes zero error covariances, where this error is systematic (e.g. due to similar wording), the question of correlated errors is one that can be tested [79, 80]. Furthermore, while correlating error terms limits the causal power of the latent factor [81], dimensional covariance between measures was of interest in the current study rather than latent disorders. We therefore included correlated error terms in the current analysis, in line with Reise et al. [59].

#### Evaluation of mental health models

Intra cluster correlations for indicator variables were calculated to assess non-independence due to sampling from schools. Since these were relatively low (.004-.067), clustering was accounted for using the *type = complex* option in Mplus, which adjusts the chi-square statistics and standard errors based on non-independence [82]. After estimating the models described in hypotheses 1–3, these were compared using chi-square difference testing: Each of the correlated factors and *S*-1 models were nested in the bifactor solution following Reise [83].

#### Explained common variance

ECV represents a ratio of variance explained by the general factor to that explained by the specific factors, while the Percentage of Uncontaminated Correlations (PUC) provides the percentage of correlations that inform on the general factor relative to the specific factors [61]. When PUC is higher (more correlations relate to the general than the specific factors), less bias is introduced by misfitting a unidimensional structure to multidimensional data. High PUC in combination with moderate to high ECV suggests that though a bifactor, multidimensional structure fits well, there is a strong case for modelling the construct as unidimensional. This is because the general factor would account for most of the variance, and factor loadings in a unidimensional model would likely be very similar to those on the general factor [62]. Reise et al. [61] suggest that PUC > .80 and ECV > .60 may be sufficient to consider unidimensionality.

#### Group differences

Gender and income measurement invariance were tested for the final model through multigroup CFA. To account for the categorical nature of the M&MS items, a three-step procedure was employed: This involved the estimation of baseline models in each subgroup separately; a configural measurement invariance model, where all loading, threshold and intercept parameters were freely estimated in both groups; and a scalar measurement invariance model where loadings and intercepts/thresholds were considered in tandem, and constrained to be equal across groups [84]. Model-based associations between latent mental health factors and gender and income were then explored via individual regression statements, rather than correlations, due to the categorical nature of the exogenous variables income and gender.

## Results

### Preliminary analysis

Gender was available for every child, ever FSM eligibility was missing for .9% of the sample, while for M&MS and CORS items, missing data ranged from .6–2.6%. Data were assumed to be missing at random, due to absence on the day of data collection, error or omission of individual items, or lack of up-to-date records from the National Pupil Database. The trivial amount of missing data confirmed that results would likely not be negatively affected by using the limited information estimator WLSMV [77].

Descriptive statistics and correlations are presented in Table 1. As expected, observed wellbeing was moderately associated with both observed mental health difficulties domains, though not with gender or family income. Family income was also not significantly associated with internalising. Externalising symptoms were inversely related to being a girl, as expected.

**Table 1:** Descriptive statistics and bivariate correlations.

Variable	1.	2.	3.	4.	5.	*M*	*SD*	Min-Max
1. Internalising	–					13.87	3.36	2–27
2. Externalising	.441*	–				8.99	2.46	1–18
3. Wellbeing	-.439*	-.329*	–			32.40	7.31	0–40
4. Gender^a^	.087*	-.149*	.026	–				
5. Income^b^	.034	.150*	-.036	.033	–			

^a^ 0 = boys, 1 = girls

^b^ 0 = never eligible for free school meals, 1 = ever eligible for free school meals.

* *p* < .01.

### Evaluation of measurement models and correlated error variances

#### M&MS

Although acceptable internal consistency was found for both M&MS subscales (externalising α = .776; internalising α = .792), preliminary CFA indicated a poor factor loading for one item (“I am shy”, λ = .291), which was consistent with other analyses [28, 69]. This item also had a low item total correlation (*r* = .257), and its removal improved internal consistency (α = .799). Furthermore, we felt this item could be interpreted as conceptually different from the others (see S1 Appendix), as it is the only one clearly linked to social functioning. The fit of the initial two-factor M&MS scale, χ^2^ (103) = 549.444, *p* < .001, RMSEA = .047 (90% CI = .043-.051), CFI = .955, TLI = .947, remained good following this item’s removal, χ^2^ (89) = 511.309, *p* < .001, RMSEA = .049 (90% CI = .045-.053), CFI = .958, TLI = .951.

Modification indices supported three pairs of correlated residuals between items with similar conceptual content and or wording. These were M&MS items 1 and 3: “I feel lonely” with “Nobody likes me”; M&MS items 5 and 6: “I worry when I am at school” with “I worry a lot”; and M&MS items 7 and 8 “I have problems sleeping” with “I wake up in the night”. The inclusion of these correlated error terms resulted in good model fit, χ^2^ (86) = 262.342, *p* < .001, RMSEA = .032 (90% CI = .028-.037), CFI = .983, TLI = .979, so this modified structure was taken forward.

#### CORS

While internal consistency for CORS was acceptable (α = .745), the model fit of a unidimensional structure was poor, χ^2^ (2) = 24.831, *p* < .001, RMSEA = .076 (90% CI = .51-.104), CFI = .976, TLI = .928. Modification indices supported the inclusion of one pair of correlated errors due to conceptual and wording overlap: CORS items 1 and 3 “how am I doing” with “how am I doing at school”. The inclusion of this error correlation substantially improved fit, χ^2^ (1) = 1.281, *p* = .258, RMSEA = .012 (90% CI = .000- .062), CFI = 1, TLI = .998, and was therefore taken forward.

### Dual-factor mental health models

**Fig 2:**
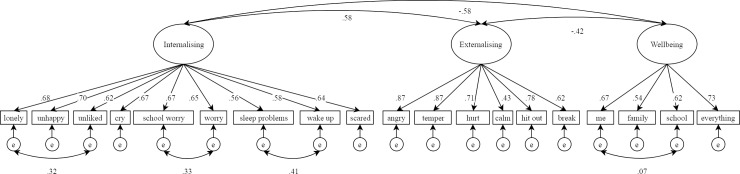
Correlated factors model results.

Hypothesis 1 was supported since the correlated factors model had excellent fit to the data (See Table 2), and significant loadings for all items (λ ≥ .43, see Fig 2). Furthermore, the estimated correlation between latent internalising and wellbeing was found to equal that between the two latent mental health difficulties dimensions (*r* = -.58). Latent externalising was also found to be substantially related to latent wellbeing, though to a lesser degree than was internalising (*r* = -.42).

**Table 2:** Fit of confirmatory factor analysis models.

Model	*χ*^2^ (df)	RMSEA(90% confidence interval)	CFI	TLI	*χ*^2^difference (df)
1. Correlated Factors	410.931** (145)	.030 (.027, .034)	.972	.967	-
2. Bifactor	321.561**(129)	.027 (.024, .031)	.980	.973	1. vs. 2. 110.742**(16)
3. *S*-1_wellbeing_	535.155**(133)	.039 (.036, .043)	.958	.946	2. vs. 3. 187.072**(4)
4. *S*-1_internalising_	407.180**(138)	.031 (.028, .035)	.972	.965	2. vs. 4. 87.311**(9)

RMSEA, Root Mean Square Error of Approximation; CFI, Comparative Fit Index; TLI, Tucker Lewis Index.

** *p* < .001.

**Fig 3:**
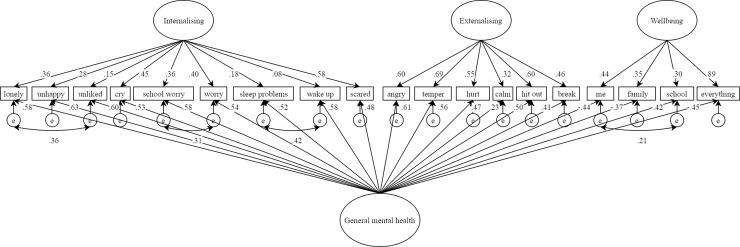
Classical bifactor model results.

Although these clear relationships were found between constructs, a unidimensional structure was not supported, as predicted in hypothesis two (PUC = .67, ECV = .55). The classical bifactor model did, however, show excellent fit to the data (see Table 2), and each item had at least one salient loading on the general or specific factor (see Fig 3). In addition to the lack of unidimensionality, inspection of the parameter estimates revealed further problems. Four internalising items had very low loadings on the specific factor (unhappy λ = .28; unliked λ = .15; sleep problems λ = .18; wakeup λ = .08), and the factor variance for internalising was also low compared to the externalising factor, which was on the same response scale (ξ = .13 versus ξ = .36). While it could be argued that internalising acted as a particularly good indicator of the general factor, we interpret this result in line with Eid et al. [56], and suggest that this is evidence of a *vanishing* factor, a result identified as consistent with the psychometric misspecification of classical bifactor solutions. Though the classical bifactor model therefore showed superior fit to other models estimated, it was rejected based on the ECV and disappearing internalising factor.

**Fig 4:**
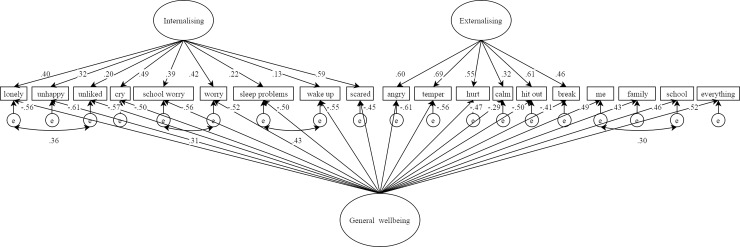
*S*-1_wellbeing_ model results.

**Fig 5:**
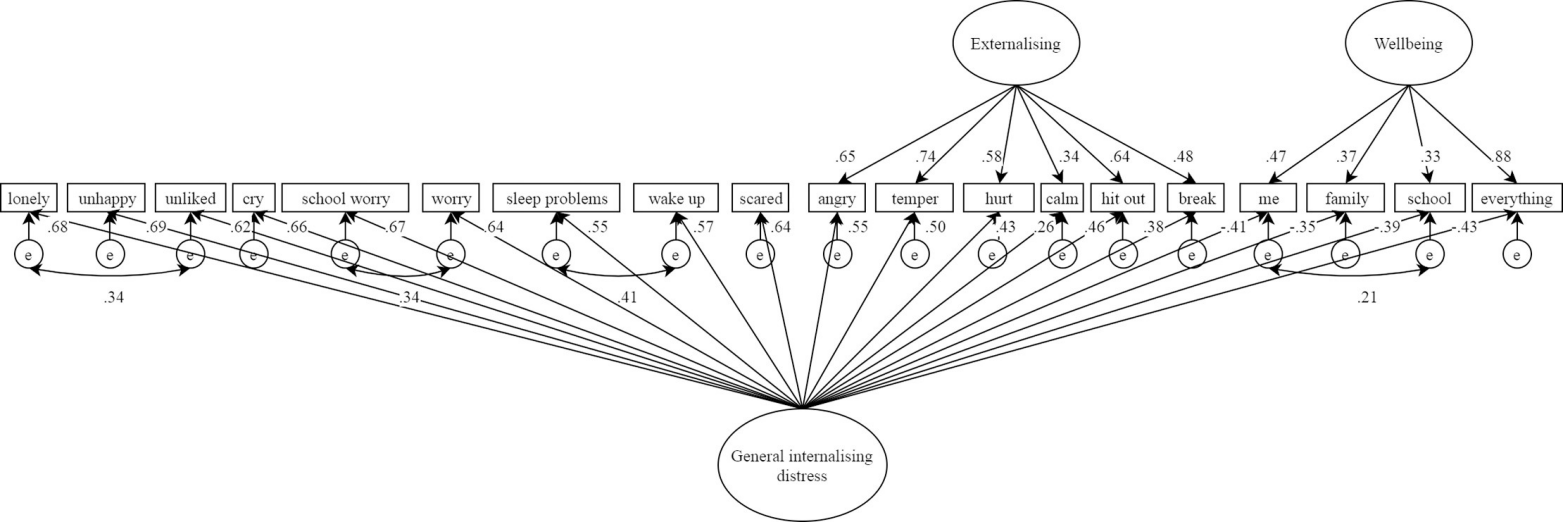
*S*-1_internalising_ model results.

Contrary to hypothesis 3, the *S*-1_wellbeing_ model was also rejected for a number of reasons. It showed inferior fit compared to the correlated model (which was less likely to overfit), the internalising factor remained relatively weak, consistent with the classical bifactor model, and the general wellbeing factor was more strongly defined by internalising than wellbeing items (see Fig 4). This suggested that general wellbeing covariance in mental health difficulties items was not a good representation of the data. In light of this, and the vanishing internalising factor found in the classical bifactor solution, post-hoc analysis of an *S*-1_internalising_ model was conducted (see Fig 5). This model showed almost identical fit to the correlated factors model (see Table 2) and unlike the *S*-1_wellbeing_ model, the general factor was this time most strongly defined by its unique items. The general factor in *S*-1_internalising_ can therefore be interpreted as modelling general internalising distress (GID) that is tapped not only by items designed to do so, but also variance of this construct captured by externalising and wellbeing items.

Difference testing was conducted between models where possible (based on number of parameters and the Nesting and Equivalence Test, NET) [85]. Of the possible comparisons, the classical bifactor model was the best as expected. It has been suggested that comparisons between models of the types we explored here should be interpreted with caution due to mathematical closeness [57]. Indeed, fit statistics revealed the correlated factors and *S*-1_internalising_ models to be extremely similar, though the latter appeared to be slightly worse based on qualitative inspection of fit statistics (this was necessary since the NET procedure revealed these models were not nested). Though the correlated factors model was therefore likely the best given its relative parsimony [74], and we recommend it be retained where possible in similar analysis, hypothesis 4 was considered in both correlated factors and *S*-1_internalising_ models since each are useful for different scenarios (see discussion below).

#### Measurement invariance testing

Invariance testing was therefore conducted on both of these models and results can be seen in Table 3. Partial measurement invariance was supported for gender in both models, with the items “I cry a lot” showing non-invariance in both, and the item “How am I doing at school” showing non-invariance in the correlated factors model. Full measurement invariance was supported for income in both models, though a small negative residual variance (-.14) was found for CORS4 (“How is everything going?”) in the ever FSM group for the *S*-1_internalising_ model. This impossible result appeared to arise from the correlated error term between the CORS items “How am I doing?” and “How am I doing at school?”, which was retained in the model since it was significant and meaningful, *r* = .26. In line with Muthén [86], the residual variance of CORS4 was fixed to zero since this parameter was non-significant (*p* = .84), and fixing this to zero did not substantially change the model fit. Since full measurement invariance is frequently seen to be untenable [87], we interpreted these results as indicating that models functioned reasonably well across the groups studied.

**Table 3:** Results of multigroup invariance testing.

Correlated Factors gender invariance					
Model	*χ*^2^ (df)	RMSEA (90% confidence interval)	CFI	TLI	*χ*^2^difference (df)
Boys baseline	295.292**(145)	.031 (.026, .037)	.970	.965	*-*
Girls baseline	258.267**(145)	.029 (.023, .035)	.980	.976	*-*
Configural	740.155**(298)	.039 (.035, .042)	.958	.952	*-*
Scalar	736.419**(345)	.034 (.030, .037)	.963	.964	82.734**(47)
Scalar M&MS4/CORS3 free	714.075**(340)	.033 (.030, .037)	.965	.965	56.103 (42), *p* = .07
***S*-1 gender invariance**					
Boys baseline	294.634**(138)	.033 (.028, .038)	.969	.962	*-*
Girls baseline	242.902**(138)	.029 (.023, .034)	.981	.977	*-*
Configural	746.480**(284)	.041 (.037, .044)	.957	.948	
Scalar	712.306**(343)	.033 (.030, .036)	.965	.965	94.405** (59)
Scalar M&MS4 free	695.876**(340)	.032 (.029, .036)	.967	.966	67.778 (54), *p* = .10
**Correlated factors income invariance**					
everfsm baseline	274.547**(145)	.032 (.026, .038)	.975	.971	*-*
neverfsm baseline	281.287**(145)	.029 (.024, .034)	.970	.964	*-*
Configural	749.155**(298)	.039 (.036, .043)	.953	.946	*-*
Scalar	698.214**(345)	.032 (.029, .036)	.963	.964	44.060 (47), *p* = .60
***S-*1 income invariance**					
everfsm baseline	268.413**(139)	.033 (.027, .039)	.975	.969	*-*
neverfsm baseline	274.571**(138)	.030 (.025, .035)	.970	.962	*-*
Configural	769.994**(284)	.042 (.038, .045)	.950	.939	*-*
Scalar	672.437**(341)	.031 (.028, .035)	.966	.966	50.095(57), *p* = .73

RMSEA, Root Mean Square Error of Approximation; CFI, Comparative Fit Index; TLI, Tucker Lewis Index; M&MS4, “I cry a lot”; CORS3, “How am I doing at school”.

** *p* < .001.

In order to estimate the association of latent mental health factors with gender and income, non-invariant items were removed from both correlated factors and *S*-1_internalising_ models [88–90] . Their removal resulted in slightly better fitting models (correlated factors without non-invariant items, *χ*^2^ = 311.847*(113), RMSEA = .030, (90% CI = .026-.034) CFI = .978, TLI = .967; *S*-1_internalising_ without non-invariant item, *χ*^2^ = 364.857*(121); RMSEA = .032 (90% CI = .028-.036); CFI = .973; TLI = .966 ) possibly due to removal of noise, and or the fact that CFI is known to be sensitive to the number of items [91]. For both models, wellbeing was not significantly associated with gender, internalising was modestly associated with being a girl, and externalising was substantially associated with being a boy (see Table 4). In line with the observed score correlations in Table 1, only externalising was significantly associated with low family income in either the correlated factors or *S*-1_internalising_ models.

**Table 4:** Gender and income associations with mental health factors.

Correlate	Internalising		Externalising		Wellbeing	
	M1	M4 (GID)	M1	M4	M1	M4
Gender	.192*	.173*	-.375*	-.612*	-.041	.116*
Income	.080	.082	.363*	.393*	-.077	-.030

M1, correlated factors model; M4, *S*-1_internalising_; GID, general internalising distress.

* *p* < .01.

## Discussion

The aim of the current study was to further our understanding of the structure of mental health difficulties and wellbeing in early adolescence, using secondary data from a large U.K. sample (*N* = 1982). Despite existing theoretical frameworks (e.g., two-continua approach) [39], the robust analysis of the measurement structure of mental health difficulties and wellbeing, and especially in younger populations, has been lacking from the extant literature. Given recent limitations pertaining to common methodological approaches, such as bifactor modeling [56–59], alternative methodologies were considered (ECV, *S*-1), and competing CFA models were estimated, which allowed for a more robust representation of the comprehensive mental health model.

Overall, unidimensionality was not supported in the current study. Instead, our results demonstrate that mental health difficulties and wellbeing are distinct but related constructs and should therefore be considered alongside each other within late childhood-early adolescent research. The simple correlated factors structure fitted the data well and revealed that wellbeing was just as related to internalising difficulties as this was to externalising symptoms. Despite the superior fit of the bifactor model, this was rejected in the current study, as the general factor explained only 55% of the total common variance. Results from the *S*-1 models further revealed that a general internalising distress factor could play an important role in all item responses. Partial gender and full income measurement invariance were established for the correlated and *S*-1_internalizing_ models. However, given that the correlated model was the most parsimonious, with a slightly better fit than that of *S*-1_internalizing_, we considered that to be the most theoretically and statistically plausible model of comprehensive mental health.

In line with previous findings [38], medium to large latent correlations were observed between wellbeing and mental health difficulties domains. The present study, however, accounted for the known distinction between childhood internalising and externalising symptoms [47], rather than conflating these as has sometimes been the case [7]. This also enabled comparison of effect sizes for estimated correlations between all latent constructs in the correlated factors model and demonstrated that wellbeing was no more dissociated from mental health difficulties constructs than these were from one another. This strengthens the idea that wellbeing may be used to *calibrate* psychopathology scores [40], and provides clear justification for the inclusion of wellbeing in mental health models.

In contrast to previous research [28, 41], we did not accept the classical bifactor solution as the final model, despite its superior fit. Since the general factor explained only 55% of the total common variance, the classical bifactor model was substantively uninterpretable, and was therefore rejected. In other words, while some previous research has suggested symptoms of mental health difficulties and wellbeing could be considered a single continuum [40], in line with hypothesis 2 our findings did not support this. We found that when internalising, externalising and wellbeing were modelled together in a large sample of early adolescents, these constructs should be treated as distinct but related factors. As suggested earlier, our choice of M&MS as a mental health difficulties measure capturing more than just negative affect, and the age of our sample, are likely to have contributed to our contrasting results. It should also be noted that this lack of support for unidimensionality is somewhat consistent with research with older adolescents [41], though in contrast to this work, we followed recent criticisms and rejected the multidimensional bifactor solution [56, 60]. This was in part facilitated by our inclusion of the ECV, which had not been considered in mental health difficulties and wellbeing bifactor models previously, and reinforces the importance of not solely relying on model fit.

Insights from stochastic measurement theory also allowed models with better defined factors to be estimated [56]. Though our hypothesised *S*-1_wellbeing_ model presented a poor fit, parameter estimates in the classical bifactor solution led to post-hoc analysis of an *S*-1_internalising_ model which explained the data well. This post-hoc analysis was conducted since internalising appeared to be weakened as a specific factor in the classical bifactor and *S*-1_wellbeing_ solutions, but showed strong loadings on the general factors in both models. In line with Eid et al. [56], we therefore considered a model in which specific internalising was removed, allowing internalising items to define the general factor. Since relatively stable general loadings were also observed across the classical bifactor and both *S*-1 models, GID covariance may have been responsible for each of these models’ general factors. Moreover, in the *S*-1_wellbeing_ model the strongest loadings on the general factor were seen for internalising, rather than wellbeing items as would be expected. Statistical comparison was not possible between the correlated factors and *S*-1_internalising_ models, and in fact it has been suggested anyway that comparison of such models is problematic, due to their mathematical closeness [57]. Nevertheless, the correlated model appeared to have slightly better fit than the *S*-1_internalising_ model, and since this was the simpler solution, we suggest that this should be preferred where possible.

This is not say, however, that the *S*-1_internalising_ model is inadmissible, as such a model would be able to address certain research questions unanswerable by the correlated factors solution. For instance, where the specific role of external correlates is of interest for particular mental health domains, as explored by Patalay et al. [7], *S*-1_internalising_ would allow researchers to estimate the effects of these on GID, externalising behaviour and wellbeing separately, while controlling for each of the other outcomes. While *S*-1_internalising_ was considered less optimal, particularly since it had more parameters, in combination with the other models and ECV results, it provides further insight into previous research. For this reason, our discussion focuses on the interpretation of both the correlated and S-1_internalising_ models.

For instance, together, our models shed light on previous findings relating to internalising. Specifically, externalising and wellbeing group factors have tended to show substantial loadings after accounting for a general factor, whereas internalising loadings have behaved differently, becoming small, sometimes insignificant, and even negative on occasion [28, 40, 41]. The *S*-1_internalising_ model could clarify this since it represents the influence of a latent internalising trait on responses to all mental health difficulties and wellbeing items. Such a structure could therefore underlie other bifactor solutions, since the consistent presence of relatively weak specific internalising suggests that this could be defining other general factors found [28, 40, 41, 56].

Theoretically GID is also consistent with the wider literature, since some of the covariance with wellbeing could be explained by the conceptual overlap (e.g. happiness and unhappiness). Covariance with externalising, on the other hand could reflect known comorbidity, which is thought to arise for a number of complex reasons, including method factors as well as cascading or predisposing effects [20, 92, 93]. Previous research has often combined internalising and externalising symptoms when considering the relationship of mental health difficulties to wellbeing [1, 3, 13]. However, our study suggests this may be problematic since both overlap and dissociation between constructs was found. It is possible that overlap at the latent level explains response patterns, and that dimensions such as those we propose should be considered rather than summed scores. While some research has categorised young people according to flourishing, languishing, etc., latent dimensional approaches could yield different results. For instance, in the *S*-1_internalising_ model it is possible that those with considerable GID show tendencies towards languishing, while those with behavioural externalising symptoms, separate from distress, could show higher wellbeing. A symptomatic but content group could therefore arise under circumstances in which the behavioural aspect of externalising is tapped as psychopathology in early adolescents who are not distressed, and therefore in turn report high wellbeing.

The estimation of both *S-*1 models in the current study, in combination with the calculation of ECV in the bifactor model, clarified the covariance structure of the items. This is namely that just over half of all common variance could be explained by a classical general factor, but that this is likely due to shared internalising variance across all items. While the current study draws on a relatively new area of work [56], current findings support the wider utility of *S*-1 models. These have not only addressed some of the concerns raised around bifactor modeling [56, 60], but also added substantive theoretical insight.

Having explored the covariance structure of mental health domains, our final aim was to shed light on their complex relationships with gender and family income. Externalising symptoms are often associated with boys, and emphasis tends to be on girls reporting higher internalising symptoms because of elevated rates in later adolescence [6, 49]. However, there is evidence that internalising symptoms also play an important role in boys’ psychopathology and externalising symptoms [67, 93]. For instance, initial lower levels of internalising were shown to predict lower levels of externalising at a later time point in both boys and girls [67].

Consistent with these studies, our results suggest only a weak association of internalising distress with gender in early adolescence. For both the correlated and *S*-1_internalising_ models internalising (at the specific level for the former, and global GID level for the latter) showed a small association with being a girl. Therefore, when specific externalising behaviour (not associated with GID) was accounted for in the *S*-1_internalising_ model, girls still showed only slightly higher levels of GID than boys. Similarly when the effect of latent internalising on externalising item responses was accounted for, the association of being a boy with externalising behaviour was notably much larger. This therefore suggests that while behavioural problems were associated with being male, this was particularly the case after controlling for GID. Furthermore, when poor behaviour (not associated with distress) was accounted for, girls still showed only slightly higher levels of internalising distress than boys. An alternative explanation for this finding could be that externalising psychopathology is entirely distinct from internalising, and remained associated with being a boy for this reason. However, five of the six externalising items had salient loadings on the GID factor (λ = .38-.64), suggesting that these items were well defined by GID, and these constructs were therefore not entirely separate.

As with gender, the associations found in the current study between mental health factors and income advance previous work which treated these factors as a single variable [7]. It was unsurprising that wellbeing did not show significant associations with low income [10]. However, it was more unexpected that only externalising was significantly and substantially related to this outcome [7], though similar conduct and emotional domains have shown stronger associations to income for the former than the latter [50]. The discrepancy in significance may therefore be due to the use of a larger sample by Fitzsimons et al. [50].

Beyond the benefits of adding *S*-1 models to understand covariance and relationships to key outcomes, the modeling approach was also strengthened by the inclusion of correlated errors. These were included to avoid overfitting in an entirely locally independent bifactor model, such that covariance beyond specific latent constructs would be absorbed by the general factor [58, 59]. These were carefully evaluated according to item content, wording and modification indices. Though inclusion of such parameters weakens the causal power of the latent trait, it is untenable to assume no relationship between conceptually similar items such as “I have problems sleeping” and “I wake up in the night” [81]. While CFA was used, the current study was somewhat exploratory, investigating the dimensionality of mental health difficulties and wellbeing, therefore allowing for relationships beyond hypothesised factors. In addition, consistent with recent calls [34], our analysis was focused at a symptom level. It therefore did not assume causal disorders, but rather considered the covariance structure of items. Nevertheless, it remains important to understand that there are associations between items beyond the latent traits modelled. As stated previously, the analysis of comprehensive mental health put forward here is not an attempt to conceptualise a definitive structure of “positive” and “ill” mental health. If such an approach were adopted, the violation of local independence would be potentially more serious in our view. Rather, our hypotheses, findings and discussion were designed to interrogate measurement assumptions routinely made for these outcomes in research with young people.

It is clear that epidemiological measures, such as those used here, can be problematic in terms of item content for local independence assumptions. While some would argue that alternative approaches to latent trait models should therefore be adopted, we feel that the robust analysis of dimensionality and covariance provided here was a key first step, before further exploration or alternative approaches considering mental health difficulties and wellbeing items together could be employed. If strong relationships between constructs had not been found in the present analysis, there would be little value in further study. It could be argued that analysis of the kind we have presented should have been employed even sooner, before analysis of correlates was considered. Our critical review of the literature and findings also suggest that categorical treatment of these outcomes can be problematic, and does not appear to be a good representation of the data. This reinforces that previous treatment of the outcomes as such [1–5, 13, 14] may lead to false conclusions.

However, it should be noted that the latent trait account we have offered may not be the only reason items covaried as they did, and that other approaches such as network analysis should be considered in future [94]. It has also been demonstrated that complex bifactor solutions can overfit data when these account for unusual response patterns [59]. Estimating the percentage of respondents who fit the model to ascertain whether complex solutions account for a minority implausible response patterns as Reise et al. [59] did, would also be pertinent to dual-factor research, given the consistent finding that a minority are neither flouring nor languishing [1–5, 13, 14].

This was the first study to our knowledge to empirically explore the structure of latent mental health difficulties and wellbeing in early adolescence. Furthermore, we employed more appropriate measures and robust approaches to bifactor modelling than those commonly used [40, 41]. Unidimensionality was not supported, but clear justification was found for the inclusion of wellbeing in mental health models, and GID was found to explain responses to all items at a salient level. This study therefore draws together and improves on school psychology dual-factor [1–5, 13, 14], and mental health bifactor research [27, 28, 30]. While the former has tended to categorically dichotomise mental health difficulties and wellbeing, and therefore lose important information [34], the latter has generally failed to account for the statistical properties of bifactor models, leading to potentially misleading conclusions [56].

Despite the use of rigorous methodology, several limitations should also be acknowledged. Firstly, the exploration of any construct is tied to the measures used, and results will inevitably vary by instrument, as already seen in the contrast between the present study and that by Böhnke et al. [40]. Though well-validated instruments were selected, replication studies should consider employing alternative measures. Similarly, constructs were assessed via self-report measures for feasibility and design reasons and as already noted, externalizing symptoms may be more accurate when reported by an adult. However, wellbeing and internalizing symptoms are likely more valid from the young person’s perspective [68]. Informant reports are also limited in that the informant (e.g. parent, teacher) typically only observes the adolescent in a single context [95]. Use of mixed informants would also likely have acted as a confound since self and informant ratings are often only weakly or moderately correlated, particularly for children and adolescents [96–98]. Though the sample size was substantial and met the recommended minimum *N*:*q* ratio (at 25.7:1), future research, particularly if more complex structural predictive components are added, should consider Monte Carlo simulations for decisions on sample size [99]. The representativeness of the sample may also be considered a limitation since poorer adolescents were overrepresented, though as stated previously, rates here were comparable to other U.K. school-based mental health research. FSM eligibility has also been criticised as a measure of socioeconomic status and proxy for family income [100], and though efforts were made to mitigate this through the use of everFSM, future studies should consider including more accurate and comprehensive measures of family income. Finally, this study used the relatively new ECV and PUC indices. While some thresholds have been recommended for these [61], further research is needed to confirm their accuracy.

## Conclusion

In the first study of its kind, early adolescents’ comprehensive mental health was explored using a large sample and robust analytical strategy. Previous research in mental health and school psychology has been extended, with our results clarifying how general factors may arise, through thorough investigation via the ECV and *S*-1 models. Clear correspondence was found between internalising and externalising symptoms, and wellbeing, and evidence suggested common GID variance was meaningfully predictive of responses to all items. This research therefore offers insight into comorbidity and dual-factor response patterns, since it suggests that common internalising may contribute across mental health domains. Given the problems with bifactor modelling in previous research, and categorical approaches often taken, our analysis provides the first robust platform from which relationships between wellbeing and mental health difficulties domains can be explored further.

## Supporting information

S1 Appendix Items of Me and My School and Child Outcome Rating Scale questionnaires. (DOCX)
