# Supplementary material for: The dimensionality and latent structure of mental health difficulties and wellbeing in early adolescence
Source: PLoS One. 2019 Feb 26;14(2):e0213018. doi: 10.1371/journal.pone.0213018 (PMC6391027; doi:10.1371/journal.pone.0213018)
Supplement: S1 Appendix — (DOCX) [file pone.0213018.s001.docx]

S1 Appendix: Items of Me and My School and Child Outcome Rating Scale Questionnaires

Each instrument is reproduced here with the items listed in the order presented in the current study.

**Me and My School**

I feel lonely

I am unhappy

Nobody likes me

I cry a lot

I worry when I am at school

I worry a lot

I have problems sleeping

I wake up in the night

I am shy

I feel scared

I get very angry

I lose my temper

I do things to hurt people

I am calm

I hit out when I am angry

I break things on purpose

**Child Outcome Rating Scale**

Me

(How am I doing?)

Family

(How are things in my family?)

School

(How am I doing at school?)

Everything

(How is everything going?)
